# 

**Published:** 1862-08

**Authors:** 


### CHICAGO MEDICAL JOURNAL.

**Terms, $2.00 per Annum.**

### CONTENTS OF THE AUGUST NUMBER.

| | Page. |
|---|---|
| Editorial and Miscellaneous | 435 |
| ORIGINAL COMMUNICATIONS. | |
| Camp Douglas | 468 |
| Foreign Correspondence. Extracts from a Letter from E. L. Holmes, M. D. | 474 |
| Camp Diarrhœa. By T. H. Walker, M. D., Chicago. | 478 |
| SELECTED. | |
| The Lessons in the Surgery of War. By Rufus King Browne, M. D., Brigade Surgeon U. S. Army, and Chief Medical Officer at Norfolk, Va | 480 |
| Clinical Notes on Intemperance and its Prevention. By Robert Druitt, M. R. C. S., London. | 483 |
| Action of Alcohol as an Aliment in Disease. | 489 |
| Pathology and Treatment of Jaundice | 491 |

